# Evolutionary and reverse engineering in *Saccharomyces cerevisiae* reveals a Pdr1p mutation-dependent mechanism for 2-phenylethanol tolerance

**DOI:** 10.1186/s12934-022-01996-x

**Published:** 2022-12-23

**Authors:** Huili Xia, Yue Kang, Zilin Ma, Cuiyu Hu, Qiao Yang, Xiaoling Zhang, Shihui Yang, Jun Dai, Xiong Chen

**Affiliations:** 1 Key Laboratory of Fermentation Engineering (Ministry of Education), Cooperative Innovation Center of Industrial Fermentation (Ministry of Education & Hubei Province), National “111” Center for Cellular Regulation and Molecular Pharmaceutics, College of Bioengineering, Hubei University of Technology, Wuhan, Hubei 430068 People’s Republic of China; 2 ABI Group, College of Marine Science and Technology, Zhejiang Ocean University, Zhoushan, 316022 Zhejiang China; 3 State Key Laboratory of Biocatalysis and Enzyme Engineering, School of Life Sciences, Hubei University, Wuhan, 430062 Hubei China

**Keywords:** Adaptive laboratory evolution, Whole genome sequencing, 2-Phenylethanol tolerance, Pdr1p mutation

## Abstract

**Background:**

2-Phenylethanol (2-PE), a higher alcohol with a rose-like odor, inhibits growth of the producer strains. However, the limited knowledge regarding 2-PE tolerance mechanisms renders our current knowledge base insufficient to inform rational design.

**Results:**

To improve the growth phenotype of *Saccharomyces cerevisiae* under a high 2-PE concentration, adaptive laboratory evolution (ALE) was used to generate an evolved 19–2 strain. Under 2-PE stress, its OD_600_ and growth rate increased by 86% and 22% than that of the parental strain, respectively. Through whole genome sequencing and reverse engineering, transcription factor Pdr1p mutation (C862R) was revealed as one of the main causes for increased 2-PE tolerance. Under 2-PE stress condition, Pdr1p mutation increased unsaturated fatty acid/saturated fatty acid ratio by 42%, and decreased cell membrane damage by 81%. Using STRING website, we identified Pdr1p interacted with some proteins, which were associated with intracellular ergosterol content, reactive oxygen species (ROS), and the ATP-binding cassette transporter. Also, the results of transcriptional analysis of genes encoded these proteins confirmed that Pdr1p mutation induced the expression of these genes. Compared with those of the reference strain, the ergosterol content of the *PDR1*_862 strain increased by 72%–101%, and the intracellular ROS concentration decreased by 38% under 2-PE stress. Furthermore, the Pdr1p mutation also increased the production of 2-PE (11% higher).

**Conclusions:**

In the present work, we have demonstrated the use of ALE as a powerful tool to improve yeast tolerance to 2-PE. Based on the reverse engineering, transcriptional and physiological analysis, we concluded that Pdr1p mutation significantly enhanced the 2-PE tolerance of yeast by regulating the fatty acid proportion, intracellular ergosterol and ROS. It provides new insights on Pdr1p mediated 2-PE tolerance, which could help in the design of more robust yeasts for natural 2-PE synthesis.

**Supplementary Information:**

The online version contains supplementary material available at 10.1186/s12934-022-01996-x.

## Introduction

As aromatic alcohol with a rose-like odor, 2-phenylethanol (2-PE) has been widely used in the perfume, cosmetics, medicine, and food industries [1]. Traditional production of 2-PE is mainly through chemical synthesis methods or by extraction from plant materials [2]. Owing to the increasing demand of consumers for natural 2-PE, the biosynthesis of 2-PE has received increasing attention [3]. Even though various strategies have been attempted to increase the concentration of 2-PE in yeast, including strain mutation and selection, optimization of medium composition, transcriptional factor engineering, promoter engineering, and metabolic modularization, the content generally remains below 6.5 g/L [4]. For example, promoter engineering and metabolic modularization were used to improve precursor transport, enhance activities of crucial enzymes, reduce by-products of the Ehrlich pathway in *S. cerevisiae* YS58, and the 2-PE concentration reached 6.3 g/L in 5 L fermenter at 120 h, which is the highest ever reported in yeast [5].

Due to the aromatic structure of 2-PE, it exhibits higher toxicity to yeast cells than ethanol. When the 2-PE concentration reached 2.5 g/L, the growth rate of *S. cerevisiae* decreased by 75% [1]. Additionally, an irregular change in cell morphology was observed in yeast subjected to 2-PE stress [6]. Transcriptome analyses suggested that 2-PE induced differences in the expression of genes associated with functions including mitochondrial activity, plasma membrane permeability, amino acid metabolism, and meiosis [7].

However, unlike ethanol, 2-PE tolerance/toxicity mechanisms have remained elusive in *S. cerevisiae*, which limits the ability to increase 2-PE tolerance by using knowledge-based rational design. Adaptive laboratory evolution (ALE) has become a powerful technique to obtain and understand new microbial phenotypes without requiring a priori knowledge of the genetic alterations [8]. Currently, ALE paired with DNA sequence and bioinformatics analysis has been used to investigate adaptive responses to many stressful environments, such as osmotic pressure, high temperature, and inhibitors [9]. Given the limited information on how 2-PE inhibits growth, ALE can help generate tolerant strains and further elucidate the genetic basis underlying tolerance phenotypes [9, 10]. In this work, we used ALE to obtain evolved strains of *S. cerevisiae* tolerant to 2-PE. The whole-genome sequence of a tolerant strain allowed us to find the genetic changes behind the 2-PE tolerance phenotypes. Reverse engineering was then performed to uncover the genetic causes for the tolerance phenotypes. A Pdr1p mutation (C862R) was shown to confer tolerance to 2-PE by multiple stress defense circuits, including changes in the fatty acid composition, ergosterol content, and intracellular ROS levels.

## Results

### Adaptive laboratory evolution and mutant screening

In order to improve the stress tolerance of S*. cerevisiae* to 2-PE and understand the stress response mechanism to serve the rational design of cell factories in the future. Herein, a microbial microdroplet culture system (MMC), an integrated platform for automated, high-throughput microbial cultivation and adaptive evolution, was used for ALE to generate evolved populations with increased tolerance to 2-PE. Before starting the 2-PE stress tolerance ALE experiment, the initial selective pressure strength was determined. The initial 2-PE pressure should be high enough to apply an adequate level of selection pressure but is not so high that it seriously impaired cells. In this study, we determined that the initial point for the ALE experiment was 0.4 g/L (Additional file 1: Fig. S1A).

**Fig. 1:**
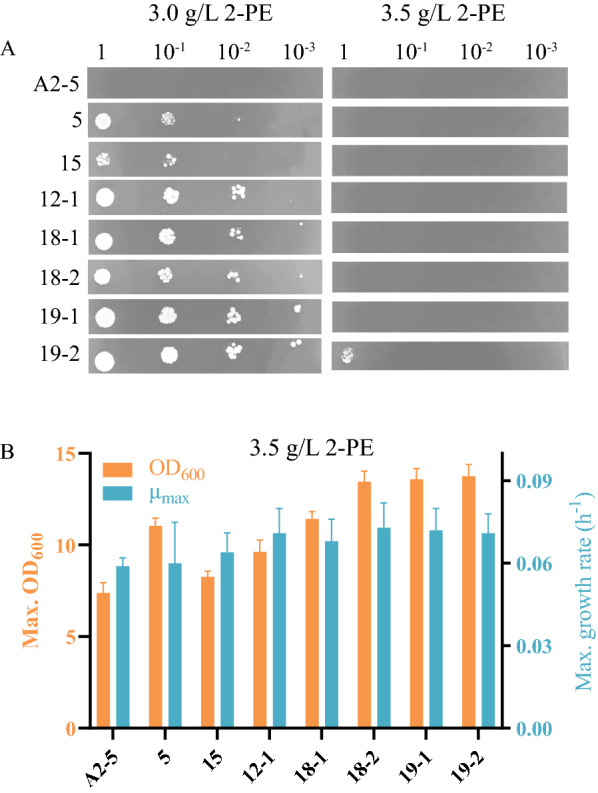
Spot assay of evolved strains in the presence of 3.0 and 3.5 g/L of 2-PE (**A**) as well as their corresponding growth rates, and maximum optical densities in the presence of 3.5 g/L of 2-PE (**B**). A2-5 strain was the parental strain and picked from the plated of *S. cerevisiae* CEN.PK113-7D. 5, 15, 12–1, 18–1, 18–2, 19–1, and 19–2 strains were the evolved strains by the ALE

The ALE was performed by using the MMC, and when the 2-PE concentration of the evolved endpoint was 8.0 g/L, the ALE experiments were terminated (Additional file 1: Fig. S1B). Due to differences in culture conditions between the MMC and plates, the 30 evolved endpoint droplets were plated on the YEPD medium containing 3.0 g/L of 2-PE, and 80 individual clones were isolated. Among these individual clones, the seven best performing clones (strains 19–2, 19–1, 18–1, 18–2, 12–1, 15, and 5) were screened to identify those with increased tolerance. As shown in Fig. 1A, the seven evolved strains had similar growth on YEPD plates without 2-PE, but only the 19–2 strain could withstand 3.5 g/L of 2-PE on YEPD plates. At 3.5 g/L of 2-PE, the growth of the 19–2 strain was better than the other strains. Its maximum OD_600_ and growth rate increased by 86% and 22%, respectively, compared to that of the A2-5 (parental strain) strain (Fig. 1B). Furthermore, it was found that: (1) under 2-PE stress, the cell morphology of the 19–2 strain remained regular, round, and plump, with a smooth surface, while the cell morphology of the A2-5 strain was irregular, elongated, and flattened, with a rough surface (Additional file 1: Fig. S2A); and (2) the fraction of cell membrane breakage (stained by PI) was decreased by 55% (Additional file 1: Fig. S2B). These results indicated that the 19–2 strain exhibited a superior tolerance and fitness performance than did the A2-5 strain.

### Whole genome sequencing of evolved mutants

**Fig. 2:**
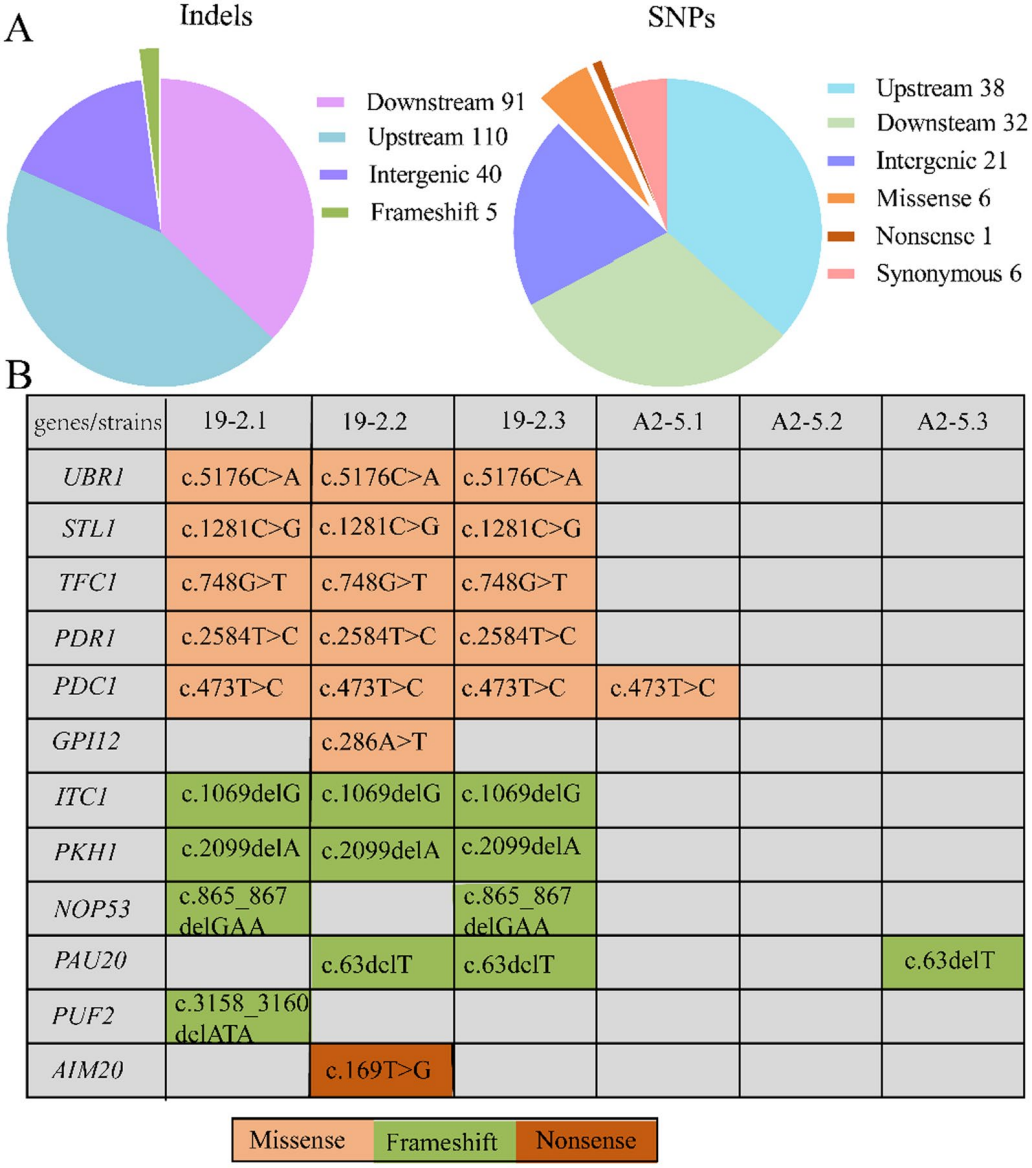
The list of all mutations (**A**), and mutations in coding sequences (**B**) detected by whole genome sequencing of the evolved 19–2 strain. Upstream and downstream mean that the mutations (indel or SNP) are located in the upstream and downstream of the coding sequencing, respectively

To explore the genetic basis underlying the acquired 2-PE tolerance phenotype, the evolved 19–2 strain and A2-5 strain (parental strain) were analyzed by whole genome sequencing, and mutations were identified in the 19–2 strain. Figure 2A shows an overview of the genetic changes in the 19–2 strain relative to the A2-5 strain. A total of 349 mutations were obtained, comprising 244 indels and 105 SNPs. The majority of mutations were located in the upstream and downstream of the coding sequencing and intergenic, only 12 mutations were located in coding sequencing (Fig. 2B).

Among the coding sequencing, *PDR1*, *PKH1*, and *PUF2* were previously shown to be associated with the structure and function of the cell wall and plasma membrane. Pdr1p is a Cys_6_Zn_2_ DNA-binding domain transcription factor, which was previously shown to confer resistance against drugs through transcriptional activation of ATP-binding cassette (ABC) transporter genes [11]. The single amino acid substitution mutations of Pdr1p elicited a gain-of-function (GOF) hyperactive phenotype, the GOF mutations invariably clustered to the central regulatory domain (CRD) of Pdr1p, such as R821S [12] and R376W [13]. In this study, a missense mutation in *PDR1* was found in the 19–2 strain, and the GOF allele (C862R) mapped to the CRD.

Furthermore, frameshift mutations in *PKH1*, *PUF2*, and *GPI12* were detected in the 19–2 strain. *PKH1* encodes a serine/threonine protein kinase that activates the MAPK cascade required for cell wall integrity, protein phosphorylation, and membrane homeostasis, and the loss-of-function *PKH1* exhibited Fe stress mitigation in yeast [14]. Puf2p is a member of the Puf family regulator that enables mRNA binding. Puf2p has shown a preference for interaction with mRNAs encoding plasma membrane-associated proteins to regulate the stability, localization, and efficiency of translation of the bound transcript [15, 16]. *GPI12* encodes an N-acetylglucosaminyl phosphatidylinositol deacetylase located in the endoplasmic reticulum, which was shown to catalyze the deacetylation of N-acetylglucosaminyl phosphatidylinositol to produce glucosaminyl phosphatidylinositol [17]. It is worth noting that *PAU20* might have a specific role in the adaptation of *S. cerevisiae* to certain environmental stresses. *PAU20* is a member of the *PAU* genes located in the subtelomeric regions of chromosomes, which were shown to evolve more quickly than the rest of the genome [18].

In addition to these five genes, other mutant genes are related to the carbon metabolism and cell growth of *S. cerevisiae*. *PDC1* encodes pyruvate decarboxylase, which converts pyruvate to acetaldehyde [19]. Mutants of the Pdc1p active site exhibited reduced production of acetaldehyde [20]. *TFC1* encodes one of two DNA-binding subunits of the yeast transcription factor τ required for cell growth [21]. Nop53p is necessary for late 60S ribosome subunit maturation and nuclear export, and it may target aberrant pre-ribosomes for surveillance and degradation pathways [22]. Itc1p is a component of the chromatin remodeling complex. Previous work demonstrated that disruption of *ITC1* led to aberrant cell morphology in *MATα* cells [23]. The mutant genes were associated with growth, carbon metabolism, and the structure and function of the cell wall and plasma membrane.

### Reverse engineering of mutations to determine causality

**Fig. 3:**
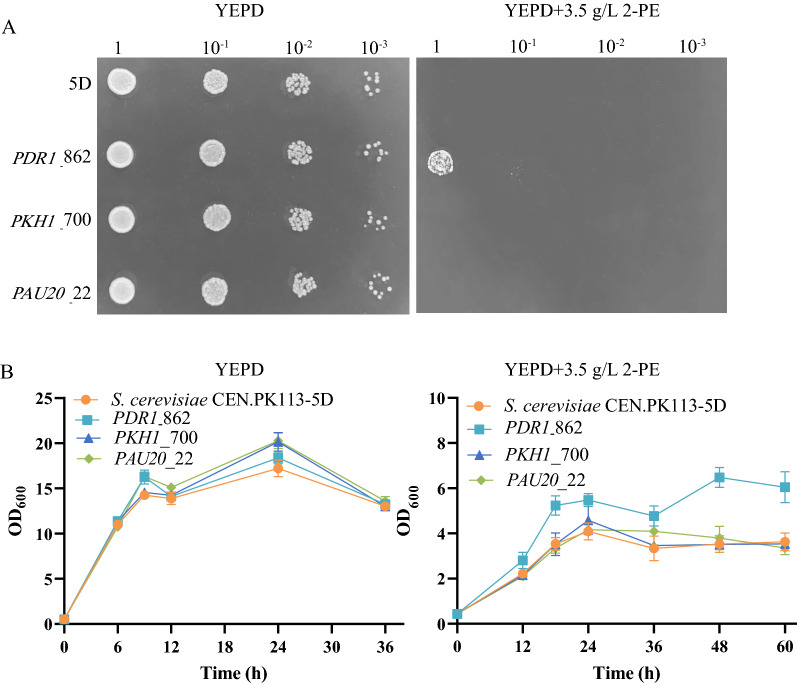
Spot assay (**A**) and cell growth (**B**) of the reverse engineered strains (*PDR1*_862, *PAU20*_22, and *PKH1*_700) and reference strain (*S. cerevisiae* CEN.PK113-5D)

*PDR1*, *PKH1*, and *PAU20* have been previously shown to be associated with the plasma membrane, stress tolerance, and stressful adaptation [14, 18, 24]. The plasma membrane functions as the biological barrier to separate the interior of cells from the external environment. It was often selected as an efficient target for increasing the stress tolerance of industrial strains [25, 26]. To test the contribution of mutations in *PDR1*, *PKH1*, and *PAU20* to the 2-PE tolerance phenotypes, each mutation was reengineered into the reference strain (*S. cerevisiae* CEN.PK113-5D), producing the engineered strains *PDR1*_862, *PKH1*_700, and *PAU20*_22. Figure 3A shows the tolerance characteristics of the engineered strains and reference strains. The engineered strains and reference strain had similar growth on YEPD plates without 2-PE, but only the *PDR1*_862 strain withstood 3.5 g/L of 2-PE on YEPD plates. At 3.5 g/L of 2-PE, the growth of the *PDR1*_862 strain was greater than the other strains, with a maximum OD_600_ 67% greater than the reference strain. The growth of *PKH1*_700 and *PAU20*_22 was similar to the reference strain (Fig. 3B). These results indicated that the mutation of *PDR1* significantly increased the tolerance of *S. cerevisiae* to 2-PE. However, there was no difference in the 2-PE stress tolerance properties between *PKH1*_700, *PAU20*_22, and the reference strain. The *PAU20* and *PKH1* mutation failed to increase the resistance to 2-PE.

**Fig. 4:**
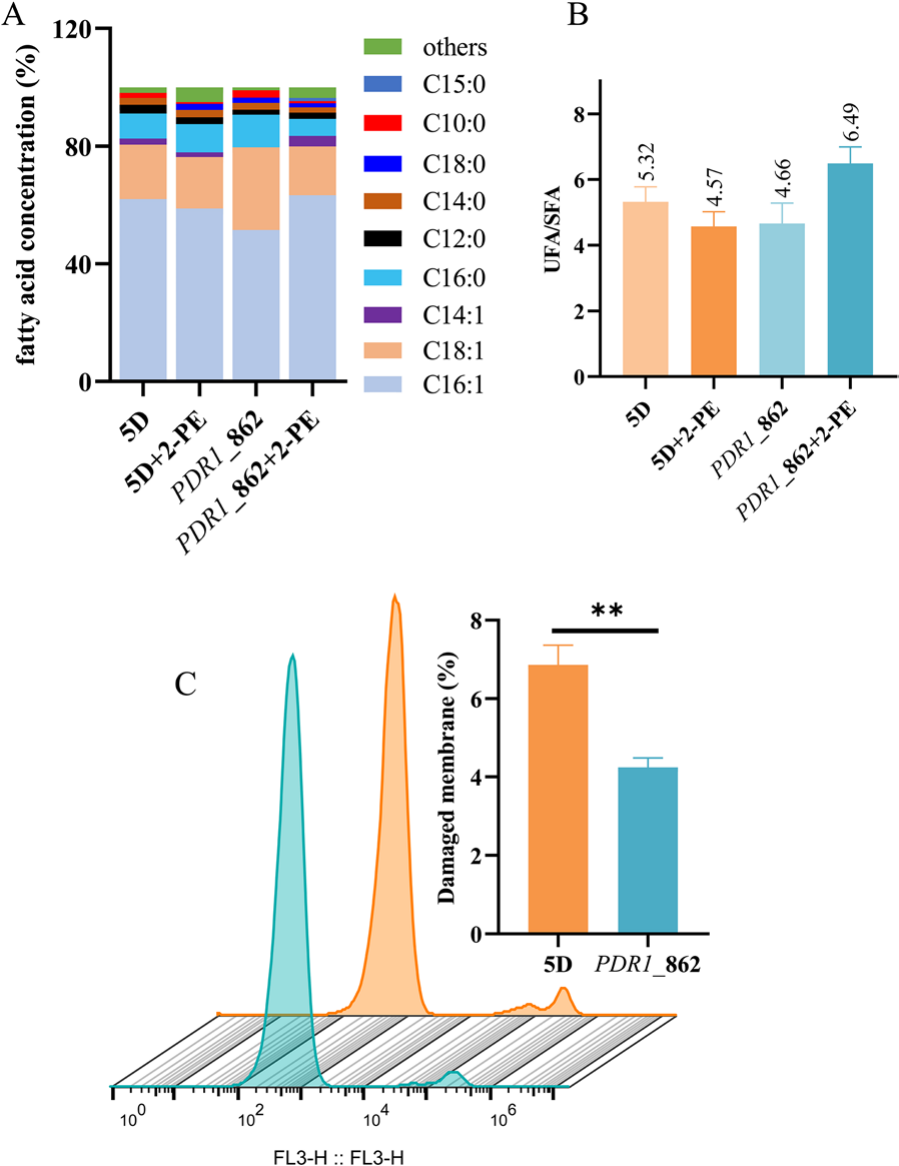
The fatty acid composition (**A**) and unsaturated fatty acid/saturated fatty acid ratio (**B**), and membrane integrity (**C**) of *PDR1*_862 strain and reference strain in the absence and presence of 3.5 g/L of 2-PE

To further investigate the effects of *PDR1* mutation on the physiological properties of the cell membrane under different 2-PE stress conditions, the fatty acid composition of the *PDR1*_862 strain and reference strain (*S. cerevisiae* CEN.PK113-5D) was analyzed. As shown in Fig. 4A, palmitoleic acid (C16:1) was the majority of the fatty acid content in reference strain. Compared to cells grown without 2-PE, the percentage of C16:1 in the *PDR1*_862 strain increased by 23%, and the unsaturated fatty acid/saturated fatty acid ratio (UFA/SFA) also increased by 39% under 2-PE stress (Fig. 4B). However, the percentage of C16:1 and UFA/SFA ratio in the reference strain cultured with 2-PE decreased by 5% and 14%, respectively, compared to the same strain cultured without 2-PE. Furthermore, under 2-PE stress, the percentage of C16:1 and UFA/SFA ratio in the *PDR1*_862 strain increased by 8% and 42%, respectively, compared to that of the reference strain.

In addition, the cell membrane integrity of the *PDR1*_862 and reference strain were further compared. As shown in Fig. 4C, in the presence of 3.5 g/L of 2-PE, the fraction of the cell membrane breakage of *PDR1*_862 strain was decreased by 38% than that of the reference strain. These results indicated that the *PDR1* mutation played an important role in modulating the fatty acid proportion to maintain membrane integrity in response to 2-PE stress.

### Pdr1p ^C862R^ plays an important role in 2-PE stress tolerance

To further investigate the underlying mechanisms of Pdr1p^C862R^ in the 2-PE stress response, we obtained 12 proteins from a network of proteins suspected to interact with Pdr1p as identified by the STRING website (http://string-db.org/) [27]. As shown in Fig. S3A, Pdr1p interacted with Pdr5p, Pdr15p, Pdr10p, Pdr12p, Pdr11p, Yor1p, Aus11p, Upc2p, and Erg11p. The proteins have roles in multidrug resistance, oxidative stress, sterol metabolism, and weak acid stress (Additional file 1: Fig. S3B) [28]. Among these proteins, Pdr5p, Pdr15p, Pdr10p, Pdr12p, Pdr11p, Yor1p, and Aus11p belong to the ATP-binding cassette (ABC) transporters, which confer resistance to various drugs and toxins [28]. Aus1p, Pdr11p, Erg11p, and Upc2p were shown to be associated with the intracellular ergosterol level [29]. Two additional genes, *CTT1* and *GSH2*, encode cytoplasmic catalase and glutathione synthase, respectively, which have shown important roles in oxidative stress [30, 31]. Taken together, we speculated that the improved 2-PE tolerance conferred by Pdr1p^C862R^ was attributed to the perturbations of the expression of all of these genes, which controlled the intracellular ergosterol content, mediated ABC transporters, and controlled intracellular ROS.

**Fig. 5:**
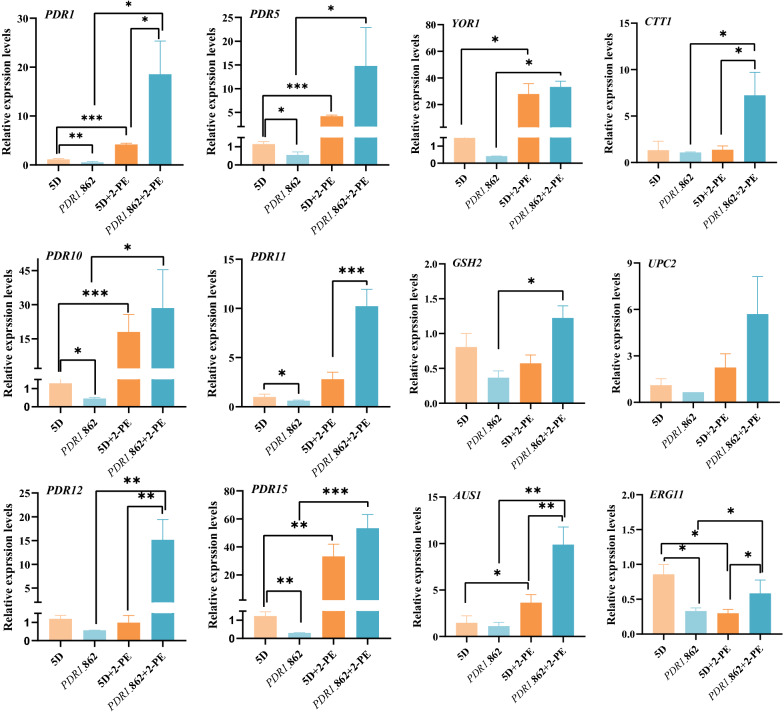
Transcription levels of the reference strain and *PDR1*_862 strain with or without 3.5 g/L of 2-PE

To verify this hypothesis, the expression levels of the respective genes were measured by real-time quantitative PCR (Fig. 5). When the reference strain exhibited stress at 3.5 g/L of 2-PE, the transcriptional levels of *PDR1*, *PDR5*, *PDR10*, *PDR11*, *AUS1*, *YOR1*, and *PDR15* were significantly upregulated compared to cells cultured without 2-PE. However, at 3.5 g/L of 2-PE, the transcriptional level of *ERG11* was significantly downregulated compared to cells without 2-PE treatment, which could block ergosterol biosynthesis. Erg11p (lanosterol 14-α-demethylase) was identified as the rate-limiting enzyme for the production of ergosterol [29]. When the *PDR1*_862 strain was cultured in 3.5 g/L of 2-PE, the transcription levels of all the genes monitored were increased compared to the same strain cultured without 2-PE and to the reference strain in the presence of 2-PE. The opposite effect was found when the *PDR1*_862 strain was cultured without 2-PE, in which the transcription levels of all the monitored genes were decreased compared to those of the reference strain also cultured without 2-PE. These results indicated that the expression of these genes was subjected to regulation by Pdr1p ^C862R^, and these genes might be the targets for 2-PE stress adaptation in response to the Pdr1p mutation.

**Fig. 6:**
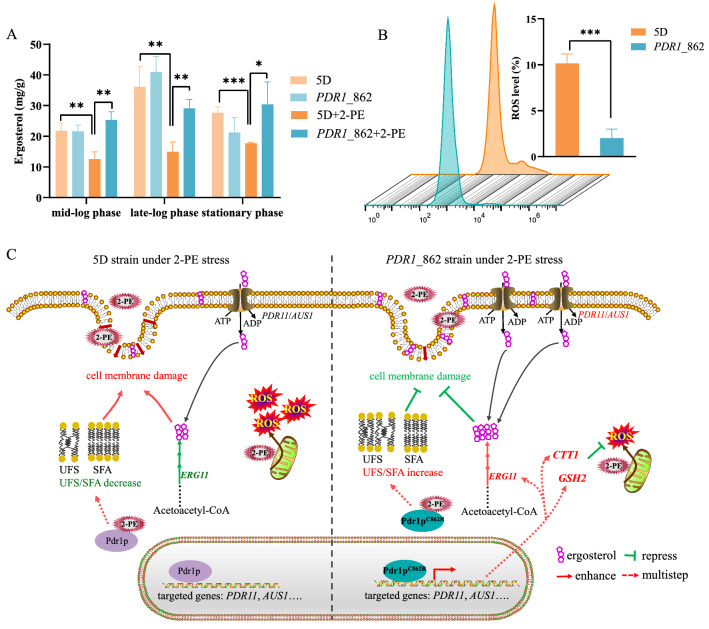
The ergosterol contents (**A**) and ROS levels (**B**) of the *PDR1*_862 strain and reference strain at 3.5 g/L of 2-PE. **C** A hypothetical working model of 2-PE tolerance mechanism

Given that the expression of *AUS1*, *PDR11*, *ERG11*, and *UPC2* was disturbed, and these genes were involved in controlling the intracellular ergosterol content, we quantified and compared the intracellular ergosterol amount in the *PDR1*_862 and reference strains. As shown in Fig. 6A, at 3.5 g/L of 2-PE, the intracellular ergosterol content of the reference strain in mid-log, late-log, and stationary phases significantly decreased by 42%, 59%, and 36%, respectively, compared to the reference strain cultured without 2-PE. 2-PE stress reduced intracellular ergosterol in the reference strain. However, at 3.5 g/L of 2-PE, compared to the reference strain, the intracellular ergosterol content of the *PDR1*_862 strain in mid-log, late-log, and stationary phases significantly increased by 101%, 94%, and 72%, which was attributed to the increase of transcription levels of *AUS1*, *PDR11*, and *ERG11*. Furthermore, the intracellular ergosterol content of the *PDR1*_862 strain in mid-log phase and stationary phase had no significant difference compared to the same strain cultured without 2-PE; only in the late-log phase was the intracellular ergosterol content significantly decreased by 29% compared to the same strain cultured without 2-PE. These results suggested that the Pdr1p mutation increased the intracellular ergosterol content to enhance 2-PE stress tolerance.

In addition, the genes *CTT1*, *GSH2*, and *PDR5* associated with ROS levels were also upregulated in the *PDR1*_862 strain under 2-PE stress. Here, ROS levels were compared in the reference strain and *PDR1*_862 strain under 3.5 g/L of 2-PE. As shown in Fig. 6B, in comparison to the reference strain, the intracellular ROS concentration of the *PDR1*_862 strain was reduced by 81%. These results indicated that Pdr1p^C862R^ regulated the intracellular ergosterol and ROS levels in response to 2-PE stress (Fig. 6C).

### Influence of the Pdr1p mutation on 2-phenylethanol production

**Fig. 7:**
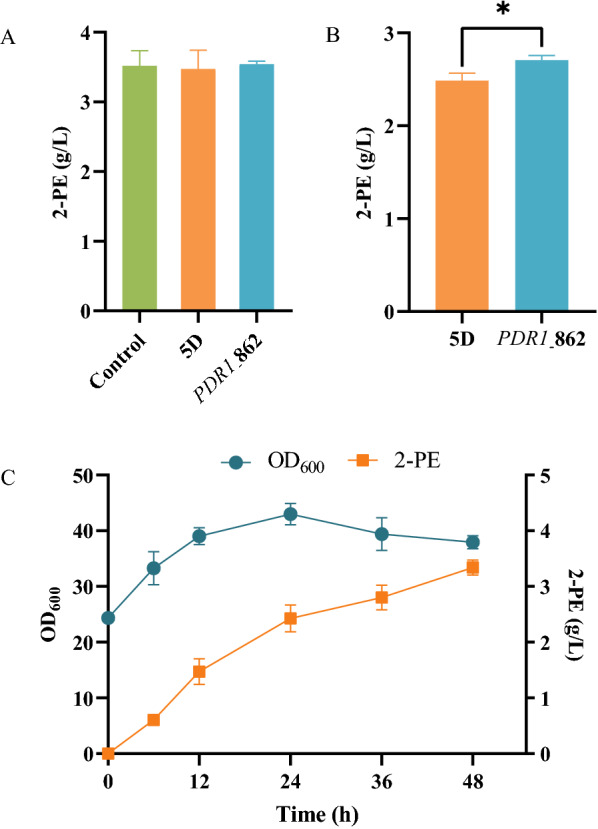
The extracellular 2-PE levels of the reference strain, *PDR1*_862 strain, and 19–2 strain in YEPD medium with 3.5 g/L of 2-PE (**A**), their 2-PE production by whole-cell bioconversion (**B**), and the fermentation characteristics of the *PDR1*_862 strain by whole-cell bioconversion (**C**)

Since the Pdr1p mutation endowed *S. cerevisiae* with improved growth performance under 2-PE stress, the *PDR1*_862 strain had the potential to be used as an industrial strain. However, not all 2-PE-tolerant strains are suitable for industrial production. If the tolerance was the consequence of degradation of 2-PE, the tolerant strain would have no value for industrial applications. In this study, under 3.5 g/L of 2-PE, the extracellular 2-PE remained unchanged, demonstrating that 2-PE was not degraded (Fig. 7A). Therefore, since the *PDR1*_862 strain was a candidate industrial strain for producing 2-PE, its ability to produce 2-PE was further evaluated. As shown in Fig. 7B, the 2-PE concentration of the *PDR1*_862 strain (2.45 g/L) by whole-cell bioconversion significantly increased by 11% over the reference strain, and the highest 2-PE concentration reached 3.34 g/L at 48 h (Fig. 7C).

### Pdr1p mutation tolerance to additional inhibitors

**Fig. 8:**
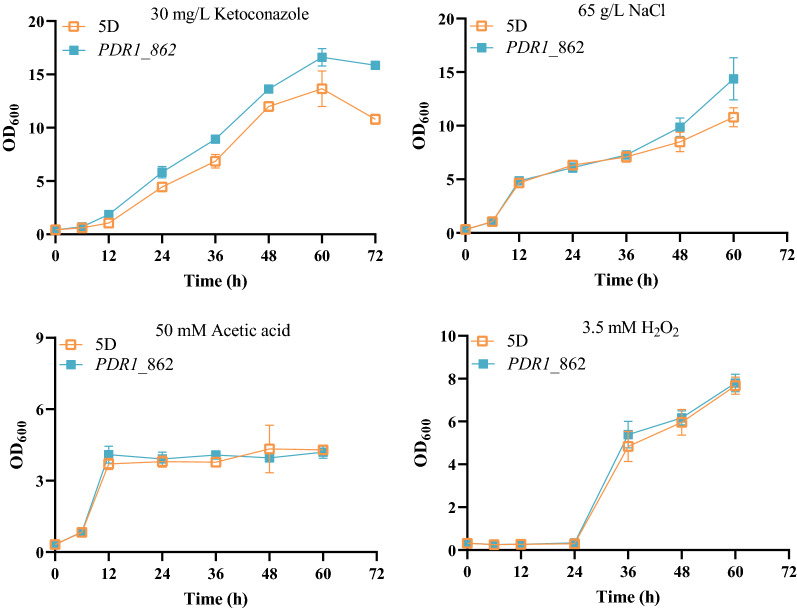
Effect of the Pdr1p mutation on the tolerance to additional inhibitors

The Pdr1p mutation increased 2-PE tolerance of *S. cerevisiae*, which led us to question if a similar effect would occur for other inhibitors. As shown in Fig. 8, there was no increase in tolerance of the *PDR1*_862 strain to 65 g/L of NaCl, 50 mM acetic acid, or 3.5 mM H_2_O_2_. However, with 30 mg/L of ketoconazole at 36 h, the OD_600_ of *PDR1*_862 was significantly increased by 30% compared to that of the reference strain. Pdr1p mutation (C862R) conferred tolerance to ketoconazole.

## Discussion

Improving the growth phenotype of *S. cerevisiae* under a high 2-PE concentration is a crucial design objective development of robust cell factories. However, the molecular mechanisms of 2-PE tolerance are poorly understood, which renders our current knowledgebase insufficient to inform such rational design. In this study, we utilized ALE to obtain the evolved 19–2 strain withstood 3.5 g/L 2-PE. Compared to many other yeasts, 19–2 strain exhibits higher tolerance to 2-PE, for example, thermotolerant *S. cerevisiae* Ye9-612 and evolved *S. cerevisiae* AD032 only tolerant 2.5 g/L and 3.2 g/L 2-PE, respectively [32, 33].

After performing whole genome sequencing and screening causal genes, we found that *PDR1* mutation (C862R) can improve fitness in the presence of 2-PE at a high level. Although a series of site mutations in the Pdr1p have been shown to improve ketoconazole, alkanes, metal ion resistance, such as Pdr1p ^R821S^, Pdr1p ^F815S^, Pdr1p ^R821H^, Pdr1p ^R376W^ [12, 13], the effect of Pdr1p mutation on the 2-PE tolerance of yeast is not clear. In this study, we firstly demonstrated that Pdr1p mutation conferred tolerance to 2-PE, and revealed that the proportion of fatty acid, intracellular ergosterol and ROS were the targets for 2-PE stress adaptation in response to Pdr1p mutation (Fig. 6C). The findings could provide new insights on Pdr1p mediated 2-PE tolerance, and help in the design of more robust yeasts for 2-PE biosynthesis. However, a precise understanding of the molecular basis underlying Pdr1p mediated 2-PE tolerance is still elusive [34]. It has been demonstrated that Pdr1p can directly bind ketoconazole [11], then activate transcription of target genes, which led us to speculate that Pdr1p directly binds 2-PE to regulate the expression of target genes. Pdr1p mutation (C862R) did also confer tolerance to ketoconazole.

Although the *PAU20* mutation could also alter the proportion of fatty acids and significantly reduce the cell membrane damage caused by 2-PE stress (Additional file 1: Fig S4), the mutation failed to improve the resistance of *S. cerevisiae* to 2-PE stress. *PAU* genes have specific roles in adaptation of yeasts to various environmental stresses, but the function of Pau protein has yet to be defined [18]. In addition, Pdr1p mutation not only conferred tolerance to 2-PE but also increased the 2-PE production. The 2-PE concentration of *PDR1*_862 strain increased by 11% than that of the reference strain, the maximum concentration of 2-PE was 3.34 g/L, which was higher than that of some yeasts, such as engineered *S. cerevisiae* S288c (2.61 g/L), *Y. lipolytica* (2.67 g/L) [35, 36]. But, the concentration of *PDR1*_862 strain has not reached maximum inhibiting levels (3.5 g/L) and the highest 2-PE production by the engineered *S. cerevisiae* YS58 (6.3 g/L). Therefore, *PDR1*_862 strain has a space to further improve the 2-PE concentration by the rational design, such as transcriptional factor engineering, efflux pump engineering, dynamic tolerance engineering.

## Conclusions

In the present work, ALE was applied to enhance the tolerance to 2-PE. As a result, a robust strain 19–2, which showed a superior tolerance and fitness performance than the parental strain. Pdr1p mutation (C862R) greatly enhanced the 2-PE tolerance of yeast, and the proportion of fatty acid, intracellular ergosterol and ROS were the targets for 2-PE stress adaptation in response to Pdr1p mutation. Meanwhile, Pdr1p^C862R^ also increased the production of 2-PE. *PAU20* and *PKH1* mutation were not directly relevant for 2-PE tolerance. In general, the data in this study revealed the Pdr1p^C862R^-dependent mechanism for 2-PE tolerance, and provides new insights on Pdr1p mediated 2-PE tolerance, which could help in the design of more robust yeasts for natural 2-PE synthesis.

## Materials and methods

### Strain and cultivation conditions

All strains, plasmids, and primers used in this study are presented in Table S1. *Escherichia coli* JM110 (dam^−^) was used for plasmid construction, and *S. cerevisiae* CEN.PK113-7D was employed for ALE experiments. The reversed-engineered strains were constructed based on the reference strain *S. cerevisiae* CEN.PK113-5D.

Selection and maintenance of plasmids in *E. coli* were performed in Luria–Bertani medium supplemented with 100 mg/L of ampicillin sodium salt. For the transformants with auxotroph markers, synthetic complete (SC) medium without uracil was used. SC medium with 1 mg/mL 5-fluoroorotic acid (5-FOA) was used for counterselection of recombinants containing the *URA3* marker. The medium components were purchased from Angel Yeast Co., Ltd. (Yichang, China). Primers were purchased from Sangon Biotech (Wuhan, China). The restriction enzyme T4 DNA ligase was purchased from Takara (Beijing, China). 2-PE was purchased from Sigma-Aldrich (St. Louis, MO, USA).

### Adaptive laboratory evolution

*S. cerevisiae* CEN.PK113-7D was cultured in SC medium and then plated on SC plates. After incubation, a single clone was selected and named A2-5. The strain A2-5 was used as the parental strain and cultured in a shake flask with SC medium. The A2-5 suspension was seeded into a microbial microdroplet culture system (MMC) [37], from which 30 droplets (2.0 μL) were generated, and 30 independent replicate droplets (30 independent lineages) were serially propagated. The concentration of 2-PE progressively increased to encourage improvement in the tolerance fitness. The detailed protocol is provided in Additional file 1.

### DNA extraction, sequencing, and analysis

The 19–2 and A2-5 strains were cultivated overnight in 5 mL of yeast extract peptone dextrose (YEPD) medium containing 3.5 g/L of 2-PE. The cells were harvested, frozen immediately in liquid nitrogen, and stored at − 80 °C. Then, the cells were sent to Shanghai Majorbio Bio-pharm Biotechnology Co., Ltd. for sequencing.

The Illumina reads of the 19–2 and A2-5 strains were mapped to the reference genome of *S. cerevisiae* CEN.PK113-7D using BWA software. The insertion-deletion (indel), single nucleotide polymorphism (SNP), structure variation, and copy number variation were identified from the aligned results by using the Haplotyper method of the GATK toolkit [38]. The A2-5 strain was used as a control strain to exclude false-positive mutations.

### Strain construction

The reverse-engineered strains with point mutations were constructed using the CRISPR-Cas9 system [39]. A 20-bp gRNA was selected near the mutation site of the *PDR1*, *PKH1*, and *PAU20* genes, and their protospacer adjacent motif (PAM) sites were inactivated using a synonymous substitution. The oligos of the gRNAs were hybridized as described previously [39], then directly ligated into linearized pML104 plasmids, and transformed into *E. coli* JM110. The recombinant plasmids (pML104-pdr1, pML104-pkh1, and pML104-pau20) were sequenced using the T3 primer to confirm the gRNA insertion into the pML104 plasmid.

The repair fragments were designed to introduce the desired mutation and inactivate the PAM site. The upstream and downstream regions of each repair fragment were amplified from *S. cerevisiae* CEN.PK113-5D genomic DNA and spliced by overlap polymerase chain reaction (PCR). Then, the repair fragments were sequenced to confirm that each mutation was successfully introduced. Finally, 1 μg of the repair fragments were transformed together with 1 μg of recombinant plasmids into *S. cerevisiae* CEN.PK113-5D cells by the lithium acetate method.^13^ Isolated colonies from the transformation were tested by PCR and confirmed by sequencing.

### Spot assay

*S. cerevisiae* was cultured in YEPD medium to log phase growth, and the cells were harvested by centrifugation (4000 × g, 5 min) and suspended with sterile water. After that, the cells were diluted to an OD_600_ of 1 with sterile water. The yeast cells in aliquots of tenfold serial dilutions were spotted on YEPD plates with or without 3.5 g/L of 2-PE supplementation and cultured at 30 °C for 2–5 days.

### Fatty acid analysis

The reference strain and mutant strains were cultivated on YEPD plates with or without 2.5 g/L of 2-PE, and 40 mg of wet yeast was used for fatty acid extraction and methylation, according to the previous study [40]. Fatty acids were analyzed by using an Agilent 6890 N gas chromatograph with MIDI Sherlock YEAST6 data analysis.

### Flow cytometry assay

The yeast was incubated to log phase in YEPD medium with different concentrations of 2-PE. The yeast cells were washed three times with PBS buffer and resuspended and diluted to an OD_600_ of 0.2. Then the cell suspension was stained with propidium iodide (PI) to evaluate membrane integrity [25] and the oxidant-sensitive probe 2′,7′-dichlorofluorescein diacetate [6] for 30 min at 4 °C in the dark. Next, the stained samples were ultrasonicated for 1 min. A BD fluorescence-activated cell sorting (FACS) flow cytometer (BD Biosciences, New Jersey, USA) was used to measure the fluorescence of the samples, with 10,000 cells analyzed for each sample. All data were analyzed by using FlowJos software (FlowJo-V10).

### Ergosterol extraction and determination

The yeast cells were cultivated in YEPD medium with or without 3.5 g/L of 2-PE, and the cells in mid-log phase, late-log phase, and stationary phase were harvested by centrifugation (4000×*g* for 5 min). The sterol was extracted according to a previously described method [41]. The ergosterol was detected by high-performance liquid chromatography (HPLC; ThermoFisher Scientific, USA) equipped with an absorbance detector set at 282 nm. The samples were analyzed using a C18 column (4.6 mm × 250 mm, 5 µm) at 25 °C. The flow rate was 1.5 mL/min, using a 97% (v/v) methanol solution as the mobile phase.

### Real-time quantitative polymerase chain reaction assay

The yeast was cultivated to log phase and harvested. Total RNA was extracted using a Total RNA kit (TIANGEN, China). cDNA was generated using HiScript II QRT SuperMix with gDNA (Vazyme Biotech, China). After that, a quantitative analysis of the mRNA was performed by using a ChamQ Universal SYBR qPCR Master Premix (Vazyme Biotech, China) on a CFX96TM Real-Time System (Bio-Rad, USA). In this study, the *ENO1* gene was used as a loading control for normalization. The relative changes in the mRNA level were calculated using the 2^−∆∆CT^ method, where CT is the threshold cycle. The primers used are listed in Additional file 1: Table S2.

### Whole-cell bioconversion

*S. cerevisiae* was incubated in YEPD for 10 h at 30 °C and 200 rpm. The cells were harvested by centrifugation (4000×*g*, 5 min) and washed by potassium phosphate buffer (pH 5.0). Then, the cell pellet was resuspended by potassium phosphate buffer. The final cell pellet was added into catalytic system (25 mL, 250 mL-flask) at an initial OD_600_ value of 20. The catalytic system consisted of 70 g/L glucose, 8.0 g/L L-Phe, 15 mg/100 mL glutamate, 20 μg/mL glutamate. The bioconversion reactions were performed at 30 °C, 200 rpm for 48 h.

### Analytical methods

The cell density (OD_600_) was measured by a spectrophotometer (752 N, China). The concentration of 2-PE and L-Phe was measured by HPLC [42]. Morphological assays of yeast cells were analyzed by a differential interference contrast microscope (DIC, ZEISS Microscopy).

### Statistics and reproducibility

The significance of groups of data was determined with a *t* test by using GraphPad Prism 8 (GraphPad Software, USA). The data analysis and graphing were performed by GraphPad Prism 8. All experiments were conducted with three biological replicates.

## Supplementary Information

**Additional file 1: Fig S1.** The adaptive laboratory evolution experimental procedures to investigate 2-PE tolerance in *S. cerevisiae*. **Fig S2.** Cell morphology of the evolved strains at 0 g/L and 3.5 g/L 2-PE and the cell membrane integrity of 19–2 strain and A2-5 strain at 0 g/L and 4.0 g/L 2-PE. **Fig S3.** The association network of Pdr1p from the STRING website and the different cellular processes they participate in. **Fig S4.** The fatty acid composition (A) and unsaturated fatty acid/saturated fatty acid ratio (B), and membrane integrity (C) of the *PKH1*_700, *PAU20*_22 strains in the absence and presence of 3.5 g/L of 2-PE. **Table S1.** Strains and plasmids used in this study. **Table S2.** Primers used in this study.

## Data Availability

The data used during the current study are available from the corresponding author on reasonable request.
