# Supplementary material for: Evolutionary and reverse engineering in Saccharomyces cerevisiae reveals a Pdr1p mutation-dependent mechanism for 2-phenylethanol tolerance
Source: Microb Cell Fact. 2022 Dec 23;21:269. doi: 10.1186/s12934-022-01996-x (PMC9789650; doi:10.1186/s12934-022-01996-x)
Supplement: Supplementary file 1 — Additional file 1: Fig S1. The adaptive laboratory evolution experimental procedures to investigate 2-PE tolerance in S. cerevisiae. Fig S2. Cell morphology of the evolved strains at 0 g/L and 3.5 g/L 2-PE and the cell membrane integrity of 19–2 strain and A2-5 strain at 0 g/L and 4.0 g/L 2-PE. Fig S3. The association network of Pdr1p from the STRING website and the different cellular processes they participate in. Fig S4. The fatty acid composition (A) and unsaturated fatty acid/saturated fatty acid ratio (B), and membrane integrity (C) of the PKH1_700, PAU20_22 strains in the absence and presence of 3.5 g/L of 2-PE. Table S1. Strains and plasmids used in this study. Table S2. Primers used in this study. [file 12934_2022_1996_MOESM1_ESM.docx]

Evolutionary and reverse engineering in *Saccharomyces cerevisiae* reveals a Pdr1p mutation-dependent mechanism for 2-phenylethanol tolerance

Huili Xia^1^, Yue Kang^1^, Zilin Ma^1^, Cuiyu Hu^1^, Qiao Yang^2^, Xiaoling Zhang^2^, Shihui Yang^3^, Jun Dai^1,2,3*^, Xiong Chen^1*^

^1^ Key Laboratory of Fermentation Engineering (Ministry of Education);

Cooperative Innovation Center of Industrial Fermentation (Ministry of Education & Hubei Province), Hubei University of Technology;

National "111" Center for Cellular Regulation and Molecular Pharmaceutics, Wuhan 430068, P.R. China.

^2^ABI Group, College of Marine Science and Technology, Zhejiang Ocean University, Zhoushan, Zhejiang, China.

^3^State Key Laboratory of Biocatalysis and Enzyme Engineering, School of Life Sciences, Hubei University, China, 430062

*Correspondence:

Xiong Chen

cx163_qx@163.com;

Jun Dai

jundai@hbut.edu.cn

Table S1 Strains and plasmids used in this study

| Strains and plasmids | Characteristics | Sources |
| --- | --- | --- |
| *S. cerevisiae* CEN.PK113-7D | *MATaURA3 HIS3 LEU2 TRP1 MAL2*-*8c SUC2* | Lab stock |
| *S. cerevisiae* CEN.PK113-5D | *MATa ura3*-*52 HIS3 LEU2 TRP1 MAL2*-*8c SUC2* | Lab stock |
| *E. coli* JM110 | dam^-^, host for plasmid construction | Takara |
| *PDR1*_862 | CEN.PK113-5D *PDR1* ^T2584C^ | This study |
| *PKH1*_700 | CEN.PK113-5D *PKH1* ^2099delA^ | This study |
| *PAU20*_22 | CEN.PK113-5D *PAU20* ^63delT^ | This study |
| pML104 | Expresses Cas9, gRNA, and *URA3* marker | Addgene Inc |
| pML104-pdr1 | Target to the *PDR1* | This study |
| pML104-pkh1 | Target to the *PKH1* | This study |
| pML104-pau20 | Target to the *PAU20* | This study |

Table S2 Primers used in this study

| **Primers** | **Sequences** |
| --- | --- |
| PDR1-gRNA F | gatcaggtataatagaacttgtctgttttagagctag |
| PDR1-gRNA R | ctagctctaaaacagacaagttctattatacct |
| PKH1-gRNA F | gatcctcccgtacctcaatcgaatgttttagagctag |
| PKH1-gRNA R | ctagctctaaaacattcgattgaggtacgggag |
| PAU20-gRNA F | gatcgtcagattgagctagagtgggttttagagctag |
| PAU20-gRNA R | ctagctctaaaacccactctagctcaatctgac |
| PDR1-U F | tatcacaagcattctcagtggccgaattca |
| PDR1-U R | cgataagcgcGagagtttgtatcTaagacaagttctattataccttttaaatctgg |
| PDR1-D F | aacttgtcttAgatacaaactctCgcgcttatcgatttttattagaacctgttc |
| PDR1-D R | ggtgctgagcgaccattgaatggcaattg |
| PKH1-U F | gatgaaggaggtagcgctttccacac |
| PKH1-U R | ttagtctattcgattgaggtacgggaggagcttgattcttttttttgtattctgtc |
| PKH1-D F | aaaaaaagaatcaagctcctcccgtacctcaatcgaatagActaataaatggtttaccg |
| PKH1-D R | tttcatctgtccgtgtcatgtttttccgc |
| PAU20-U F | gatgagatatggaggatatgtgaggtgctaaatgagcatc |
| PAU20-U R | agagtggtagttgcgaagcagaagcagtagcagcgat |
| PAU20-D F | actgcttctgcttcgcaactaccactctagctcaatctga |
| PAU20-D R | ccctagtttgcgatagtgtagataccatctttggatagagccttg |
| T3 | gcaattaaccctcactaaagg |

**Adaptive laboratory evolution**

**Determined the initial selective pressure of 2-PE**

The native tolerance of the parental strain A2-5 to 2-PE was determined by monitoring the OD_600_ in SC medium with different concentrations of 2-PE (Fig S1A). In the presence of 0.4 g/L 2-PE, the OD_600_ was similar with that in the absence of 2-PE (the control). When the concentration of 2-PE increased to 0.9 g/L, the OD_600_ was decreased by 23% compared with the control. The initial 2-PE pressure is high enough to apply an adequate level of selection pressure, but is not too high that seriously impairs cells. Therefore, we determined that the initial point for ALE experiment would be 0.4 g/L.

**Adaptive laboratory evolution**

Microbial microdroplet culture system (MMC) is an integrated platform for automated, high-throughput microbial cultivation and adaptive evolution (Jian et al., 2020; Jian et al., 2022). Pick the single colony of A2-5 from agarose plate and cultivate it in shake flask to prepare the initial microbial solution. Then the initial microbial solution was injected into the reagent bottle, conduct the adaptive evolution in MMC.

Adaptive evolution involves continuous automatic sub-cultivation through droplet splitting and fusion, the concentration of 2-PE progressively increased to encourage improvement in the tolerance fitness. During the evolution process, 30 droplets (2.0 μL) were generated, which were equivalent to 30 shake flask cultivation units, and the inoculation volume was set to 15% in each sub-cultivation. The cultivation time of each sub-cultivation cycle was 24 h (Jian et al., 2022). Adaptive evolution experiments were terminated after producing 77 transfers, and the 2-PE concentration of evolved endpoint was 8.0 g/L (Fig S1B). Due to differences in culture conditions between MMC and plate, the 30 evolved endpoint droplets were plated on the YEPD medium containing 3.0 g/L 2-PE, 80 individual clones were isolated.


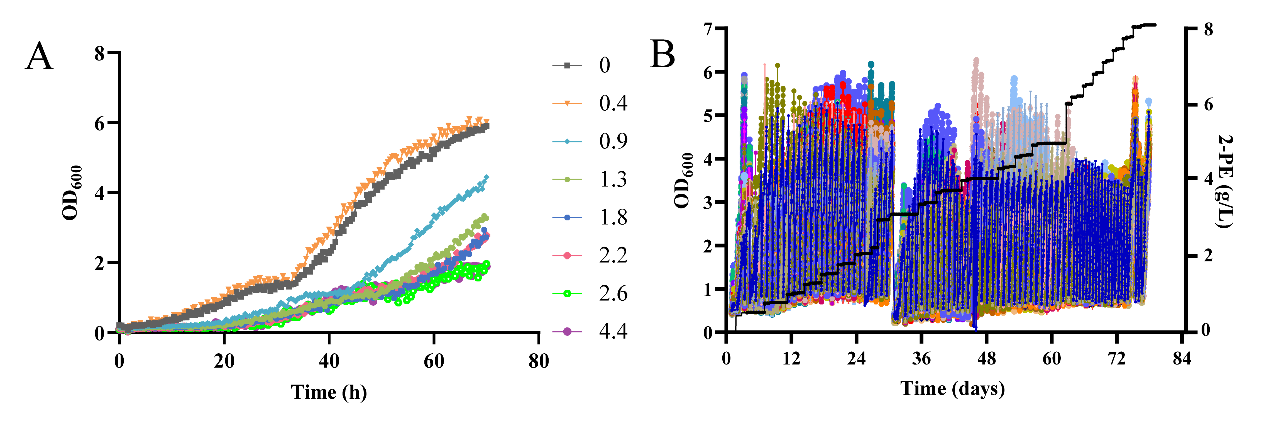


Fig S1 The adaptive laboratory evolution experimental procedures to investigate 2-PE tolerance in *S. cerevisiae*. (A) Pre-screening the growth of *S. cerevisiae* was determined in the presence of 2-PE at different concentrations to determine the optimum starting point for adaptive laboratory evolution experiment. (B) Adaptive laboratory evolution *S. cerevisiae* was serially transferred (77 transfers) into fresh media with increasing concentration of 2-PE by the microbial microdroplet culture system.


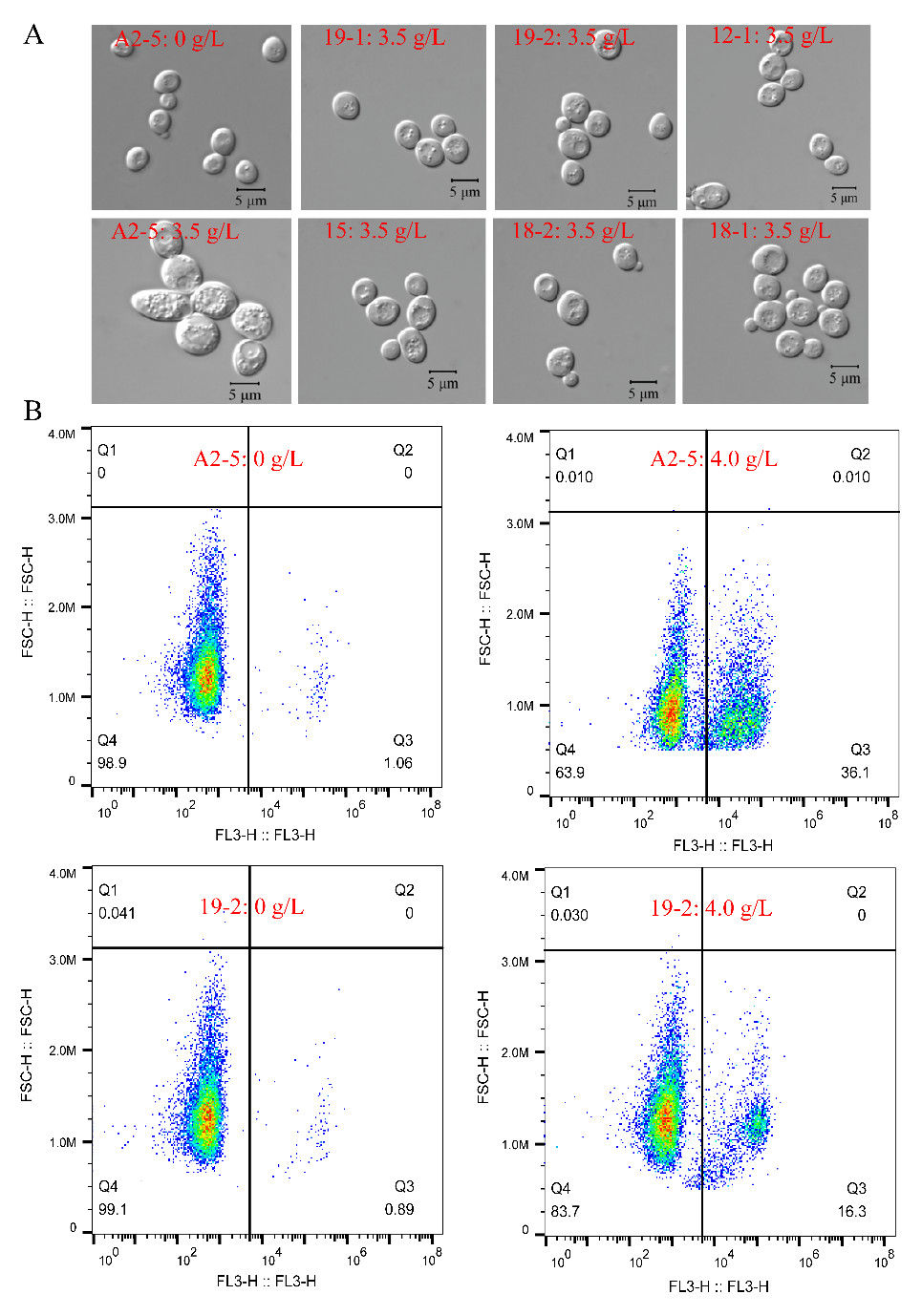


Fig S2 Cell morphology of the evolved strains at 0 g/L and 3.5 g/L 2-PE (A) and the cell membrane integrity of 19-2 strain and A2-5 strain at 0 g/L and 4.0 g/L 2-PE (B).


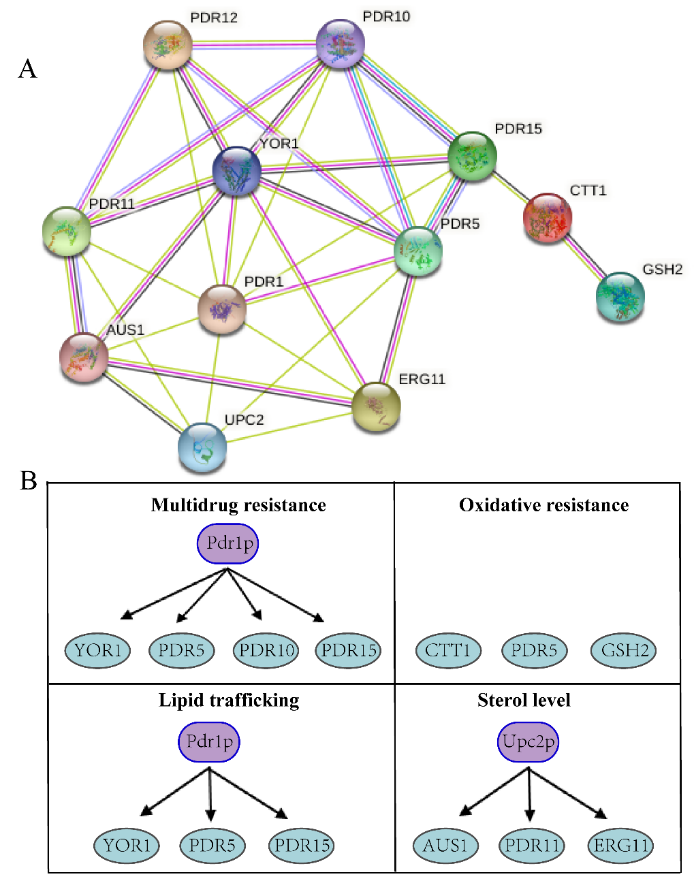


Fig S3 The association network of Pdr1p from the STRING website (A) and the different cellular processes they participate in (B).


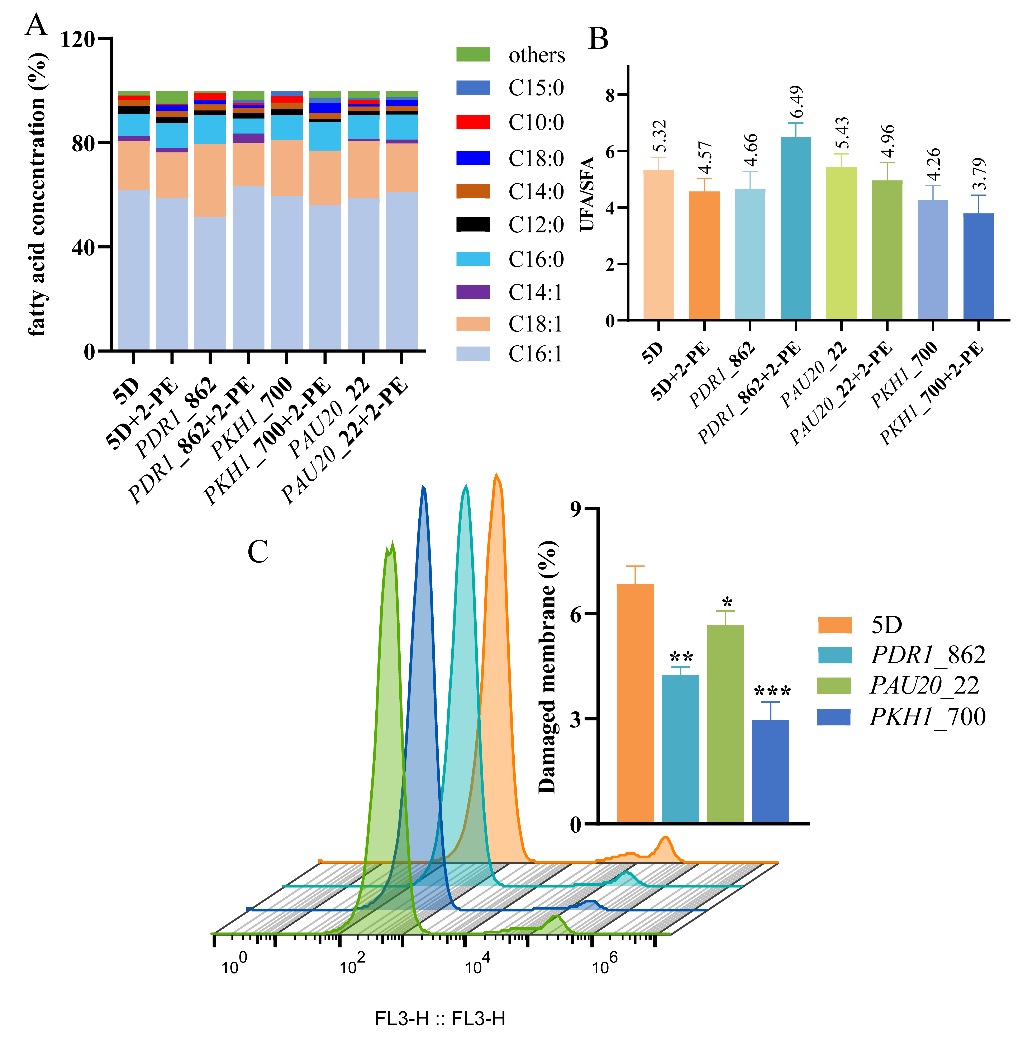


Fig. S4 The fatty acid composition (A) and unsaturated fatty acid/saturated fatty acid ratio (B), and membrane integrity (C) of the *PKH1*_700, *PAU20*_22 strains in the absence and presence of 3.5 g/L of 2-PE.

**Availability of data**

All data generated or analyzed during this study are included in this published article. The assembly and raw sequencing data have been deposited in GenBank under BioProject accession PRJNA849701, SAMN29127043 for genome assembly data, and SRR19666547 for genomic PacBio sequencing data.

**References**

Jian, X., Guo, X., Wang, J., Tan, Z. L., Xing, X. H., Wang, L., Zhang, C., 2020. Microbial microdroplet culture system (MMC): An integrated platform for automated, high-throughput microbial cultivation and adaptive evolution. Biotechnol Bioeng. 117**,** 1724-1737. doi: 10.1002/bit.27327.

Jian, X., Guo, X., Wang, J., Tan, Z. L., Xing, X. H., Wang, L., Zhang, C., 2022. Automated microbial cultivation and adaptive evolution using microbial microdroplet culture system (MMC). Journal of visualized experiments : JoVE. 18. doi: 10.3791/62800.
